# Severe Acute Respiratory Syndrome Coronavirus 2 among Asymptomatic Workers Screened for Work Resumption, China

**DOI:** 10.3201/eid2609.201848

**Published:** 2020-09

**Authors:** Xiaoyu Han, Xiong Wei, Osamah Alwalid, Yukun Cao, Yumin Li, Li Wang, Heshui Shi

**Affiliations:** Union Hospital, Tongji Medical College, Wuhan, China (X. Han, O. Alwalid, Y. Cao, Y. Li, H. Shi);; Hubei Province Key Laboratory of Molecular Imaging, Wuhan (X. Han, O. Alwalid, Y. Li, H. Shi);; Wuhan Pingan Healthcare Diagnostic Center, Wuhan (X. Wei, L. Wang)

**Keywords:** respiratory infections, severe acute respiratory syndrome coronavirus 2, SARS-CoV-2, SARS, COVID-19, coronavirus disease, zoonoses, viruses, coronavirus, Wuhan, China

## Abstract

After the outbreak in Wuhan, China, we assessed 29,299 workers screened for severe acute respiratory syndrome coronavirus 2 by reverse transcription PCR. We noted 18 (0.061%) cases of asymptomatic infection; 13 turned negative within 8.0 days, and 41 close contacts tested negative. Among 6 contacts who had serologic tests, none were positive.

As the population of Wuhan, China, returns to work, asymptomatic cases of severe acute respiratory syndrome coronavirus 2 (SARS-CoV-2) are being discovered among workers receiving health checkups for work resumption. Previous studies have shown that asymptomatic cases can be a public health threat and might lead to another outbreak (*1*,*2*). However, little is known about the clinical characteristics of asymptomatic infections. We report on cases of asymptomatic SARS-CoV-2 infection among persons during work resumption screening in Wuhan.

At Wuhan Pingan Healthcare Diagnostic Center, we reviewed 29,299 asymptomatic persons who were screened for SARS-CoV-2 by reverse transcription PCR (RT-PCR) and 22,633 asymptomatic persons tested for SARS-CoV-2 antibodies during March 13–April 25, 2020. Throat swab specimens were tested for SARS-CoV-2 by using Real-Time Fluorescent-PCR Kits (DAAN GENE Co., LTD, https://www.en.daangene.com; Appendix). We used colloidal gold-based immunochromatographic strip assay, Novel Coronavirus (SARS-CoV-2) IgM/IgG Antibody Detection Assay (Vazyme Biotech Co. Ltd., http://vazyme.bioon.com.cn) to perform antibody testing (Appendix). We recorded the demographic features, exposure history, RT-PCR and serology results, and imaging reports at the time of testing. We obtained follow-up data from persons screened by telephone.

Among 29,299 persons screened by RT-PCR, we confirmed 18 (0.061%) cases of SARS-CoV-2 infection. Of 22,633 persons tested for SARS-CoV-2 antibodies, 617 (2.7%) cases had positive IgG but negative IgM; 196 (0.87%) cases had positive IgG and IgM; and 40 (0.18%) cases had negative IgG but positive IgM.

The median age of 18 asymptomatic case-patients (10 male, 8 female) was 30.5 years (Table). Six (33.3%) cases had clear contact history with a confirmed case of SARS-CoV-2 infection. The median cycle threshold (C_t_) values on the day of first positive RT-PCR were 38.2 (C_t_ range 37.2–39.3) for ORFa1b gene and 38.1 (C_t_ range 36.81–38.5) for N gene (Table). All antibody tests were obtained on the day of first positive RT-PCR except in 1 case (obtained 6 days later). Half (7/14) the cases had negative IgM and IgG; the other half had positive IgG but negative IgM results (Table). Among 8 case-patients who had computed tomography imaging of the chest, none had remarkable findings. We closely observed the cases for 3–41 (median 16.5) days; 13 cases had negative RT-PCR within a median of 8 (range 3–14) days (Table), and none had symptoms. Among 41 close contacts, all had 2 consecutive negative RT-PCR tests >24 hours apart. Among 6 contacts who had serologic tests, none had positive results.

**Table:** Clinical characteristics of asymptomatic persons with detected severe acute respiratory syndrome coronavirus 2 infection, Wuhan, China*

Age, y/sex	Occupation		Underlying condition	Interval, d†		Exposure source	RT-PCR	C_t_ ORFa1b	C_t_ N	Interval, d‡	IgM	IgG	Chest CT	Interval, d§	Follow-up, d
29/M	Office worker		N	70		Family	+, –, –	37	38	5	–	+	Normal	0	41
32/M	Journalist		N	54		Bus passenger	+, +, –	39	38	9	–	+	Normal	0	41
27/M	Journalist		N	NA		Unclear	+, –	35	32	3	–	–	Normal	0	39
31/F	Researcher		N	61		Family	+, –, –, –, –	36	38	7	–	–	NA	NA	23
27/M	Office worker		N	75		Family	+, –, –, –	33	35	3	–	+	Normal	0	21
32/F	Housewife		N	NA		Unclear	+, –, –	38	37	14	–	–	NA	NA	17
37/F	Office worker		N	22		Family	+, –, –, –	40	39	8	NA	NA	NA	NA	16
57/M	Logistics dispatcher		Asthma	NA		Unclear	+, –, –	39	37	8	NA	NA	Normal	10	16
25/M	Banking		N	NA		Unclear	+, –, –	38	38	8	NA	A	NA	NA	16
26/M	Marketing		N	NA		Unclear	+	39	38	NA	–	–	NA	NA	17
23/M	Marketing		N	NA		Unclear	+, –	38	38	13	–	–	Normal	5	17
26/F	Office worker		N	NA		Unclear	+, –	38	40	14	NA	NA	NA	NA	17
60/F	Cleaner		N	NA		Unclear	+	37	37	NA	–	+	NA	NA	13
65/M	Cleaner		COPD	NA		Unclear	+	39	39	NA	–	–	Normal	0	13
28/F	Office worker		N	NA		Unclear	+	38	39	NA	–	+	NA	NA	8
33/F	Office worker		N	30		Family	+, –	39	37	12	–	+	Normal	0	14
30/F	Office worker		N	NA		Unclear	+, –	39	38	10	–	+	NA	NA	13
62/M	Worker	Hypertension			NA	Unclear	+	39	39	NA	–	–	NA	NA	3

*COPD, chronic obstructive pulmonary disease; CT, computed tomography; C_t_ ORFa1b, RT-PCR cycle threshold for the ORFa1b gene; C_t_ N, RT-PCR cycle threshold for the N gene; N, no; NA, not available; +, positive; –, negative. †Days between last exposure and initial positive RT–PCR. ‡Days between first positive and first negative RT-PCR results §Days between first positive RT-PCR and CT scan.

According to Wuhan Municipal Health Commission (*3*), 275,400 RT-PCR tests were performed for universal screening during April 9–15, 2020. Among those, 182 (0.066%) asymptomatic persons were identified as SARS-Cov-2–positive, which is consistent with our study. Half the cases in our study showed negative IgM and IgG at the time of positive RT-PCR, suggesting recent infections (<14 days). Seven (50%) cases in our study had positive IgG but negative IgM, indicating a late stage infection, 4 of which had a long interval of exposure (30–75 days). In addition, 13 cases had negative RT-PCR assays <8 (range 3–14) days, suggesting a favorable prognosis for persons with asymptomatic infections.

Epidemiologic, virologic, and modeling evidence support the possibility of SARS-CoV-2 transmission from persons who are presymptomatic (SARS-CoV-2 detected before symptoms onset) or asymptomatic (never develop symptoms) (*4*). None of the 18 asymptomatic persons in our study developed symptoms. Recent reports showed that the viral load of SARS-CoV-2 infections in persons with no or mild symptoms was similar to the viral load of symptomatic patients (*5**,**6*), which could contribute to rapid transmissions (*5*). However, other studies demonstrated that asymptomatic patients had a lower viral load than symptomatic and presymptomatic patients (*7*,*8*,*9*), which might indicate less transmissibility from asymptomatic persons. The median cycle threshold values for the 18 cases were 38.2 for ORFa1b gene and 38.1 for the N gene, indicating a relatively low viral load. In addition, all 41 close contacts of the asymptomatic case-patients tested negative by RT-PCR. Possible explanations for this finding include that: the asymptomatic infected persons had relatively low viral load and were less infectious; that asymptomatic persons did not have clinical symptoms, such as sneezing or coughing, that could cause virus spread; and that, due to the strict isolation and preventive measures taken in Wuhan for >3 months, the population was generally protected from the spread of infection by mask-wearing and self-quarantine.

Our report has limitations. Our sample size of asymptomatic cases is small, and follow-up was short. Recall bias of exposure history is another limitation; in the absence of clear symptom onset, asymptomatic persons might be less likely to accurately recall exposures than persons with symptoms. Finally, that the study took a place during the post-peak period of the epidemic in Wuhan, so contacts could have been seropositive already; those tested were seronegative, but most contacts did not have serologic testing.

In conclusion, as the population returns to the workplace, asymptomatic SARS-CoV-2–infected persons could be among workers. Although we did not detect transmission among 41 contacts of persons who were SARS-CoV-2–positive, such transmission cannot be excluded. Therefore, continued testing, self-quarantine, and mask-wearing should be encouraged to reduce the risk for additional outbreaks.

Appendix Laboratory methods used to detect severe acute respiratory syndrome corona virus 2 antigens and antibodies among asymptomatic persons returning to work and their contacts in Wuhan, China.
