# Supplementary material for: Severe Acute Respiratory Syndrome Coronavirus 2 among Asymptomatic Workers Screened for Work Resumption, China
Source: Emerg Infect Dis. 2020 Sep;26(9):2265–7. doi: 10.3201/eid2609.201848 (PMC7454110; doi:10.3201/eid2609.201848)
Supplement: Appendix — Laboratory methods used to detect severe acute respiratory syndrome corona virus 2 antigens and antibodies among asymptomatic persons returning to work and their contacts in Wuhan, China. [file 20-1848-Techapp-s1.pdf]

Article DOI: https://doi.org/10.3201/eid2609.201848

Severe Acute Respiratory Syndrome Coronavirus 2 among Asymptomatic Workers Screened for Work Resumption, China

Appendix

Reverse Transcription-PCR (RT-PCR) for Severe Acute Respiratory Syndrome Coronavirus 2 (SARS-CoV-2)

We performed real-time fluorescent PCR analysis on ABI 7500 (Thermo Fisher Scientific, https://www.thermofisher.com). The throat swab specimens were tested for SARS-CoV-2 by using Real-Time Fluorescent-PCR Kits (DAAN GENE Co., LTD, https://www.en.daangene.com). The coincidence rate of negative reference products (–/–) is 10/10 and all are negative; the coincidence rate of positive reference products (+/+) is 5/5.

The coefficient of variation (CV, %) of the precision cycle threshold (C_t) value is $\leq$5.0%. The minimum detection amount is $\leq$1 × 103 copies/mL. We did not detect cross reaction with human coronavirus (HKU1, OC43, NL63 and 229E), SARS coronavirus, MERS coronavirus (HKU1, OC43), other viruses, or human genomic DNA.

Method for SARS-CoV-2 Antibody Assays

We tested for SARS-CoV-2 IgM/IgG antibodies by using colloidal gold-based immunochromatographic strip assay, Novel Coronavirus (SARS-CoV-2) IgM/IgG Antibody Detection Assay (Vazyme Biotech Co. Ltd., http://vazyme.bioon.com.cn). The tested specimen (serum/plasma) diffuses upward by capillary force at the loading end and passes

through the marker pad. The SARS-CoV-2 IgM antibody and IgG antibody in the sample combine with the recombinant antigen colloidal gold to form a colloidal gold-labeled antigen-test IgM complex and a colloidal gold-labeled antigen-test IgG complex diffusing into nitrocellulose membrane. The strip includes 3 indicator lines, T1 for IgM, T2 for IgG, and C indicating control.
